# Recursive ensemble feature selection provides a robust mRNA expression signature for myalgic encephalomyelitis/chronic fatigue syndrome

**DOI:** 10.1038/s41598-021-83660-9

**Published:** 2021-02-25

**Authors:** Paula I. Metselaar, Lucero Mendoza-Maldonado, Andrew Yung Fong Li Yim, Ilias Abarkan, Peter Henneman, Anje A. te Velde, Alexander Schönhuth, Jos A. Bosch, Aletta D. Kraneveld, Alejandro Lopez-Rincon

**Affiliations:** 1 Tytgat Institute for Liver and Intestinal Research, AGEM, Amsterdam UMC, University of Amsterdam, Amsterdam, The Netherlands; 2 Unidad de Citogenética, Hospital Civil de Guadalajara “Juan I. Menchaca”, Guadalajara, Jalisco Mexico; 3 Department of Clinical Genetics, Amsterdam UMC, University of Amsterdam, Amsterdam, The Netherlands; 4 Division of Pharmacology, Utrecht Institute for Pharmaceutical Sciences, Faculty of Science, Utrecht University, Utrecht, The Netherlands; 5 Genome Data Science, Faculty of Technology, Bielefeld University, Bielefeld, Germany; 6 Department of Psychology, University of Amsterdam, Amsterdam, The Netherlands; 7 Department of Medical Psychology, Amsterdam UMC, University of Amsterdam, Amsterdam, The Netherlands; 8 Department of Data Science, Julius Center for Health Sciences and Primary Care, University Medical Center Utrecht, Utrecht, The Netherlands

**Keywords:** Diagnostic markers, Machine learning, Genetics research

## Abstract

Myalgic encephalomyelitis/chronic fatigue syndrome (ME/CFS) is a chronic disorder characterized by disabling fatigue. Several studies have sought to identify diagnostic biomarkers, with varying results. Here, we innovate this process by combining both mRNA expression and DNA methylation data. We performed recursive ensemble feature selection (REFS) on publicly available mRNA expression data in peripheral blood mononuclear cells (PBMCs) of 93 ME/CFS patients and 25 healthy controls, and found a signature of 23 genes capable of distinguishing cases and controls. REFS highly outperformed other methods, with an AUC of 0.92. We validated the results on a different platform (AUC of 0.95) and in DNA methylation data obtained from four public studies on ME/CFS (99 patients and 50 controls), identifying 48 gene-associated CpGs that predicted disease status as well (AUC of 0.97). Finally, ten of the 23 genes could be interpreted in the context of the derailed immune system of ME/CFS.

## Introduction

Myalgic encephalomyelitis/chronic fatigue syndrome (ME/CFS) is a complex disorder characterized by a persistent and debilitating fatigue that lasts for at least six months. According to the 1994 Fukuda case definition, ME/CFS includes four or more of the following symptoms: memory or concentration impairment, sore throat, tender glands, muscle pain, multi-joint pain, headaches, unrefreshing sleep and post-exertion malaise. The latter implies that fatigue symptoms worsen upon minimal mental or physical efforts^1^. Three types of ME/CFS can be distinguished, based on the medical history: 1) post-infection related, 2) (auto)immune disease related, and 3) of unknown origin. The worldwide prevalence of ME/CFS is approximately 0.76–3.28%^2^. Spontaneous recovery is less than 5% and current treatment options are considered suboptimal. ME/CFS is thus a chronic disorder that severely impacts patients’ quality of life. The etiology remains elusive, although ME/CFS appears to have a heritable component^3^ with genome-wide association studies identifying multiple risk loci^4,5^. Recent evidence suggests that non-genetic factors like infections^6^ can induce epigenetic changes^7,8^ that might be involved in etiology as well. Currently, there is no prognostic or diagnostic test available^9,10^.

Several studies focused on mRNA expression to find biomarkers that support diagnosis and better understanding of etiology. Unfortunately, the results show little consistency. Fang et al.^11^ found 164 mRNAs that were significantly differentially expressed in peripheral blood mononuclear cells (PBMCs) from fatigued versus non-fatigued participants ($n=167$). Presson et al.^12^ restructured this data and recoded fatigue severity for the 167 participants.They selected 118 participants for an Integrated Weighted Gene Co-expression Network Analysis (IWGCNA) that identified 20 candidate genes related to ME/CFS severity. In other cohorts, Gow et al.^13^ found 366 differentially expressed mRNAs in PBMCs of eight post-infectious ME/CFS patients versus seven healthy controls and Nguyen et al.^14^ identified 176 mRNAs in whole blood of adolescents (18 healthy vs. 29 patients). Between those 366 and 176 mRNAs, measured in slightly different cell populations, only ten overlapped. Finally, Byrnes et al.^15^ found no differential expression in peripheral blood leukocytes (PBLs) of 44 monozygotic twin pairs discordant for ME/CFS. In the results from four additional differential expression studies in PBLs, only one gene, *MSN* overlapped between two of them^15^.

Consideration of epigenetic factors may help resolve some of the inconsistencies. Epigenetics performs transcriptional regulation in a mitotically heritable fashion, yet can be influenced by non-genetic factors^16^. It encompasses a wide variety of modifications that do not change the DNA sequence, but are thought to affect the accessibility and hence readability thereof. One modification is DNA methylation, the addition of a methyl group to the fifth carbon of cytosine. This process has been described predominantly for cytosines followed by guanine residues (CpG)^17^. An increased concentration of CpGs is often observed in the promoter region of genes, where such clusters are called CpG islands^18^. Promoter methylation is typically inversely correlated with the expression of the associated gene, where a high density of methylated CpGs correlates with lower mRNA expression and vice versa^19,20^. De Vega et al. have performed differential methylation analyses on PMBCs of ME/CFS patients^21–23^ and reported between 1,192 and 12,608 differentially methylated CpG sites, related to 826 to 5,544 annotated genes. Using another microarray method with more probes, Trivedi et al.^24^ found 17,296 differentially methylated CpG sites related to 6,368 genes. To the best of our knowledge, no study has yet integrated mRNA expression data and DNA methylation data of ME/CFS.

In light of the preceding discussion, we sought to integrate publicly available mRNA expression and DNA methylation data to innovate biomarker research in ME/CFS. To this end, we made use of recursive ensemble feature selection (REFS), a classification pipeline which, when applied in near-analogous settings, has proven to reliably shed light on the corresponding relationships^25^. REFS was used to identify a robust mRNA expression signature that could differentiate between cases and controls. Subsequently, we investigated whether genes found to be differentially expressed in ME/CFS were indicative of differential DNA methylation as well. To equate methods and results of previous studies, we also compared the performance of REFS to IWGCNA and univariate analyses. Finally, we sought to interpret the biological function and relevance of the genes identified by REFS as candidate biomarkers for ME/CFS.

## Results

### mRNA expression feature selection

**Figure 1:**
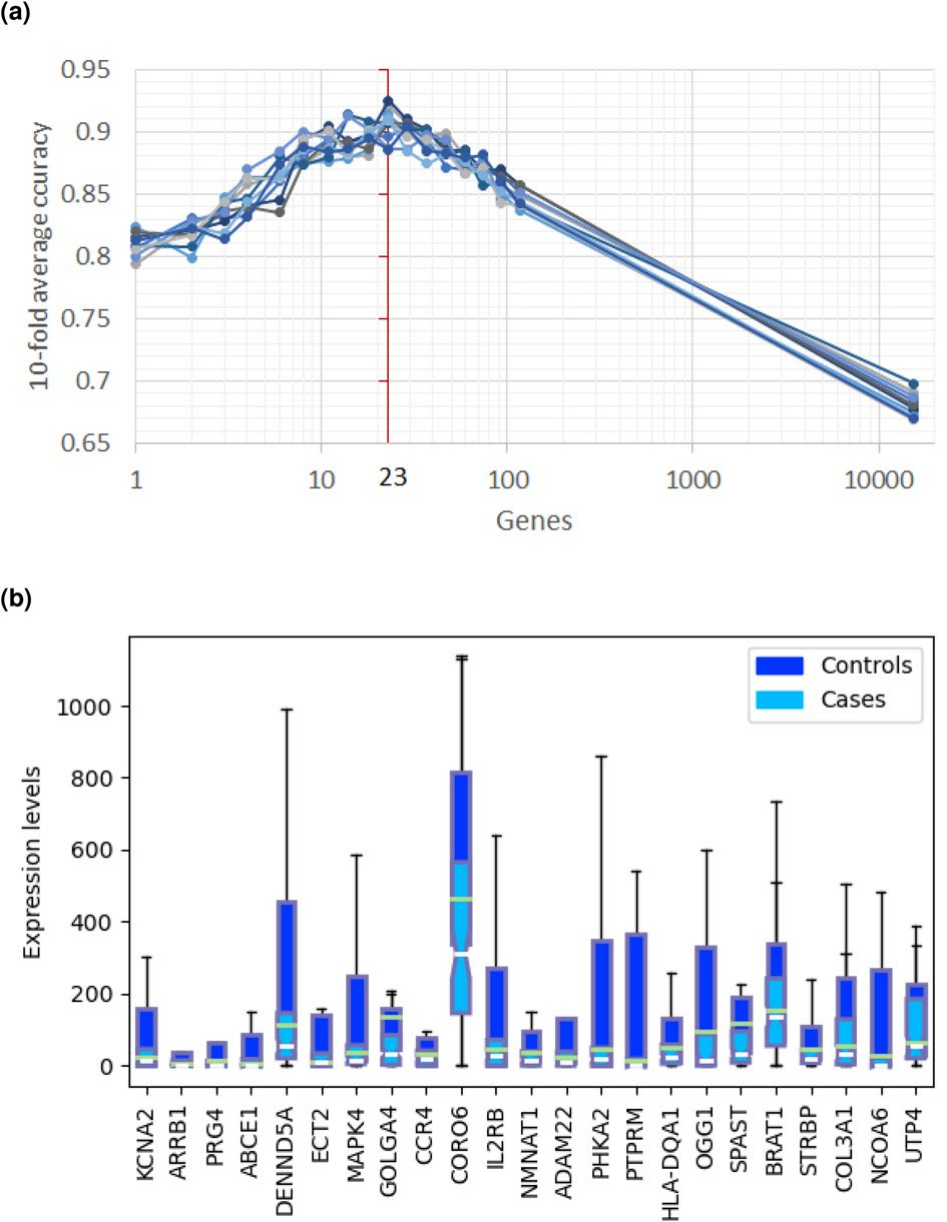
Results of the REFS algorithm run ten times on the mRNA expression data of 118 samples from the CAMDA dataset. (**a**) The optimal number of predictor genes to distinguish 93 cases from 25 controls was 23 (red vertical line). (**b**) mRNA expression levels of the 23 predictor genes for 93 cases (light blue) and 25 controls (dark blue) in box-and-whisker plots. Outliers were omitted for visualization purposes.

We ran the REFS algorithm ten times on the CAMDA mRNA expression dataset containing 118 samples and the algorithm found the optimal number of predictor genes to be 23 (Fig. 1a; Supplemental Table S1). Multivariate analysis of variance (MANOVA)^26^ indicated a statistically significant difference between healthy controls and ME/CFS patients ($F (23, 95) = 5.15$, $p < .0001$; Wilk’s $\lambda = 0.445$, R-squared (uncentered) = 0.555). mRNA expression of all 23 predictor genes was down-regulated in ME/CFS patients (Fig. 1b). Next, we compared our results with IWGCNA and univariate feature reduction using $\chi ^2$, which were used by Presson et al.^12^ and Byrnes et al.^15^, respectively, to identify genes that were associated with ME/CFS.

**Figure 2:**
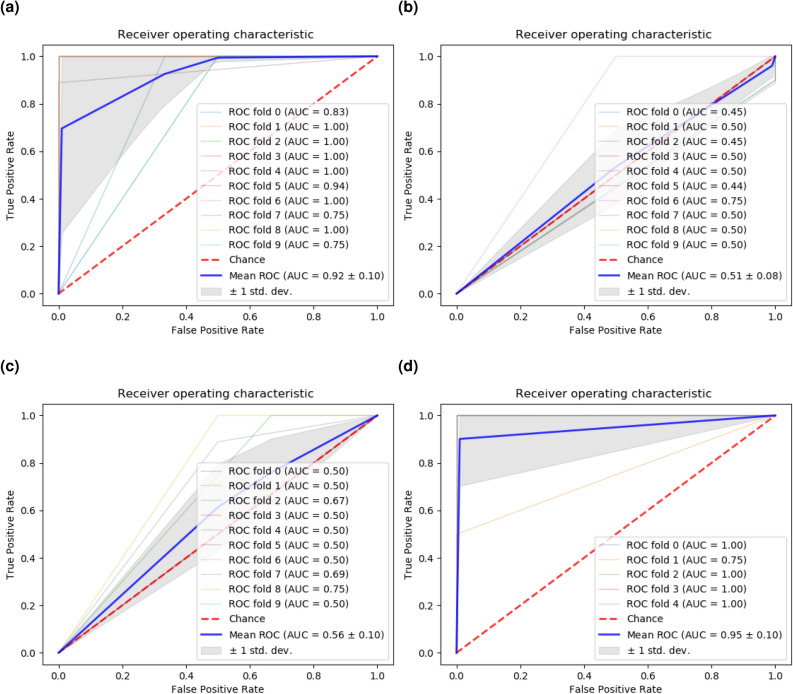
ROC curves for (**a**) REFS, (**b**) IWGCNA, and (**c**) univariate analysis applied to the same mRNA expression data of 118 samples from the CAMDA dataset. (**d**) ROC curve for the validation of the obtained 23-gene signature on a separate dataset (GSE14577). The 5-fold cross validation was performed with the eighteen genes available in GSE14577.

IWGCNA constructs a co-expression network and prioritizes modules of genes based on their association with a phenotype in combination with the presence of a disease-associated genetic variant. Essentially, IWGCNA selects the most important genes by $p-value$ score. Presson et al.^12^ reported 20 candidate genes based on mRNA expression data from the CAMDA dataset, the same dataset in which we found 23 predictor genes using REFS. The results of Presson et al. and our REFS algorithm could thus be directly compared. Univariate feature selection reduces the number of features using univariate score metrics, in this case, $\chi ^2$. For the comparison, we applied the sci-kit learning implementation^27^ to the CAMDA dataset, and selected features according to the highest $\chi ^2$ scores with a fixed $k=23$. Next, we calculated the accuracy and the associated receiver-operating characteristic (ROC) curve for all three algorithms. The REFS algorithm had an average accuracy of 91.57% differentiating controls and patients with ME/CFS globally, with the Passive Aggressive classifier showing a 95.87% accuracy using only the 23 candidate genes (Table 1). Moreover, the area under the curve (AUC) of the ROC curve for REFS was 0.92 (Fig. 2a), which is considered outstanding^28,29^. The compared methods were only slightly better than chance (IWGCNA: AUC = 0.51 (Fig. 2b), univariate analysis: AUC = 0.56 (Fig. 2c). Comparing the genes of interest each of the three methods returned, we found that REFS outperformed both IWGCNA and univariate analysis based on the ROC curve and every performance classifier (Table 1).

**Table 1:** Classification accuracy of the 23 predictor genes obtained with REFS, the 20 genes obtained with IWGCNA, and the 23 genes obtained with univariate analysis based on the same mRNA expression data of 118 samples from the CAMDA dataset

Classifier	REFS		IWGCNA		$\chi ^2$		mRNA validation		DNA methylation	
	$\mu$	$\sigma$	$\mu$	$\sigma$	$\mu$	$\sigma$	$\mu$	$\sigma$	$\mu$	$\sigma$
Gradient boosting (n_estimators=300)	0.898	0.0647	0.7417	0.0975	0.7801	0.0775	0.8111	0.1641	0.7852	0.1023
Random forest (n_estimators=300)	0.8575	0.0714	0.7298	0.0576	0.799	0.0459	0.9333	0.0943	0.8181	0.0867
Logistic regression	0.9595	0.0533	0.7138	0.0913	0.8052	0.0530	0.9333	0.0943	0.9457	0.042
Passive aggressive	0.9587	0.0675	0.6057	0.1744	0.8151	0.0693	0.9333	0.0943	0.98	0.0306
SGD	0.9595	0.0533	0.6264	0.0659	0.7564	0.0914	0.9333	0.0943	0.9324	0.0437
SVC (linear)	0.9421	0.0754	0.7472	0.0848	0.7962	0.0408	0.9333	0.0943	0.9667	0.0683
Ridge	0.9023	0.0691	0.7214	0.0858	0.8143	0.0616	0.9333	0.0943	0.9733	0.0327
Bagging (n_estimators=300)	0.8478	0.0495	0.7144	0.0841	0.7801	0.0940	0.8778	0.0875	0.8119	0.0586
Average	0.9157	0.0630	0.7001	0.0927	0.7933	0.0667	0.9111	0.1022	0.9017	0.0581

Classification accuracy of eighteen of the 23 predictor genes applied to a validation mRNA dataset (GSE14577), and of 48 predictor CpGs obtained with REFS based on merged DNA methylation data. $\mu$, mean; $\sigma$, standard deviation.

### Cross platform validation

We applied the resulting mRNA expression signature to a separate mRNA dataset (GSE14577) as a cross platform validation. Due to the small sample size ($n=15$), we did not perform feature reduction, but applied the 23 candidate genes directly to the dataset. As the data was measured with a different platform, not all genes overlapped with the CAMDA dataset. Using the DAVID gene functional tool^30^, we retrieved eighteen genes. After training the eighteen-gene based model through 5-fold cross validation, we achieved a global classification accuracy of 91.11% differentiating controls and patients with ME/CFS (Table 1) with an ROC AUC of 0.95 (Fig. 2d).

### DNA methylation feature selection

**Figure 3:**
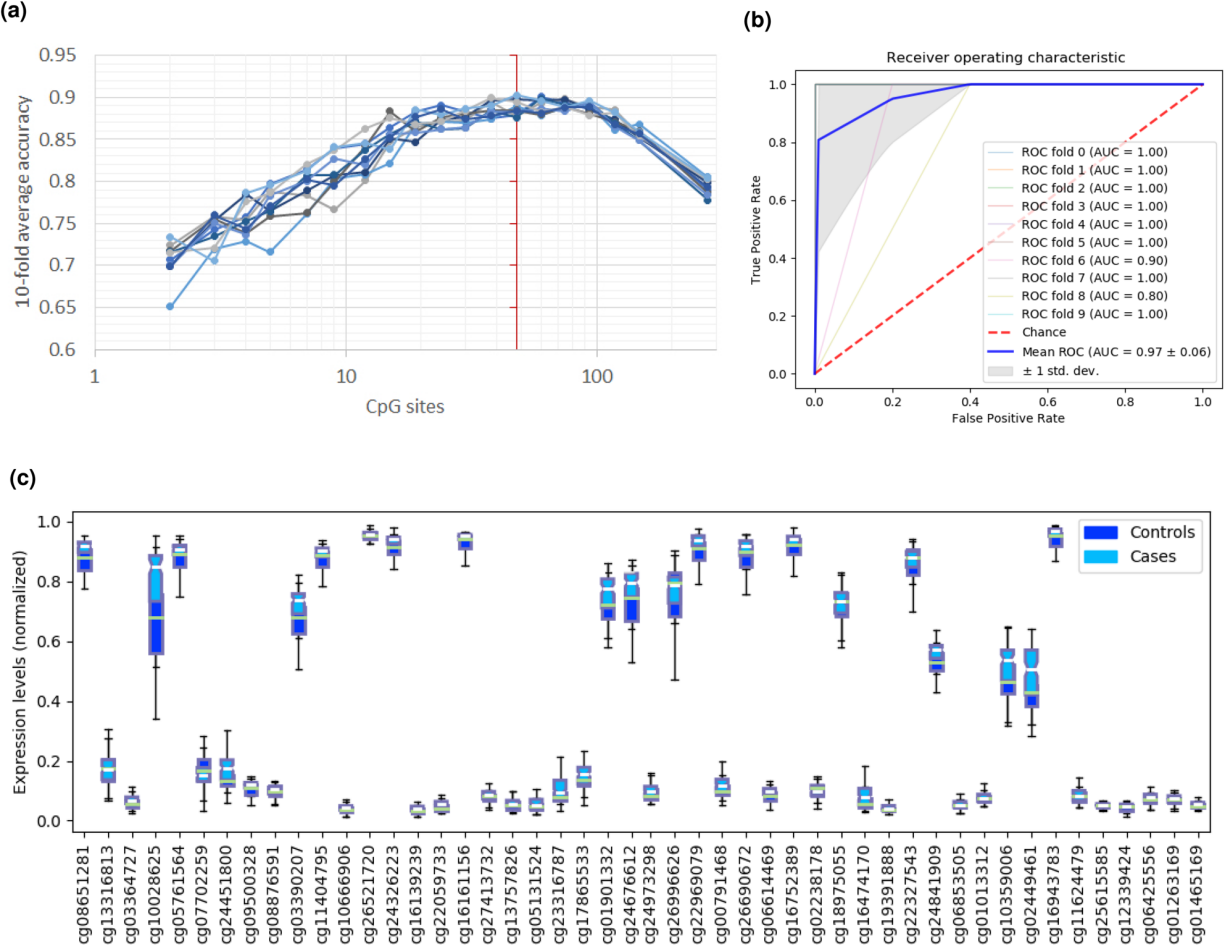
Results of the REFS algorithm run ten times on the merged DNA methylation datasets restricted to 278 probes associated with the 23 candidate genes. (**a**) The optimal number of predictor CpGs to distinguish 99 cases from 50 controls was 48 (red vertical line). (**b**) ROC curve of the 48 predictor CpGs. c) DNA methylation levels (normalized using *Standard* scaler) of the 48 predictor CpG sites for 99 cases (light blue) and 50 controls (dark blue).

We reduced the number of CpG probes to 278 by selecting for probes associated with the 23 predictor genes. Of these 278 CpGs, REFS identified 48 CpGs as predictive of ME/CFS after ten runs on the combined methylation data (Fig. 3a). MANOVA analysis showed a statistically significant difference between healthy controls and ME/CFS patients ($F (48, 101) = 50.6783$, $p < .0001$; Wilk’s $\lambda = 0.034$, R-squared (uncentered) = 0.960). The 48 CpGs reached a global accuracy of 90.17% distinguishing patients and healthy controls (Table 1). When comparing CpG methylation between cases and controls, most CpG sites showed enhanced methylation in ME/CFS patients (Fig. 3c). Finally, the calculated ROC curve had an AUC of 0.97 (Fig. 3b), pointing to a clear distinction between patients and controls when applying the 48 predictor CpG sites.

### Biological interpretation of the gene signature

**Figure 4:**
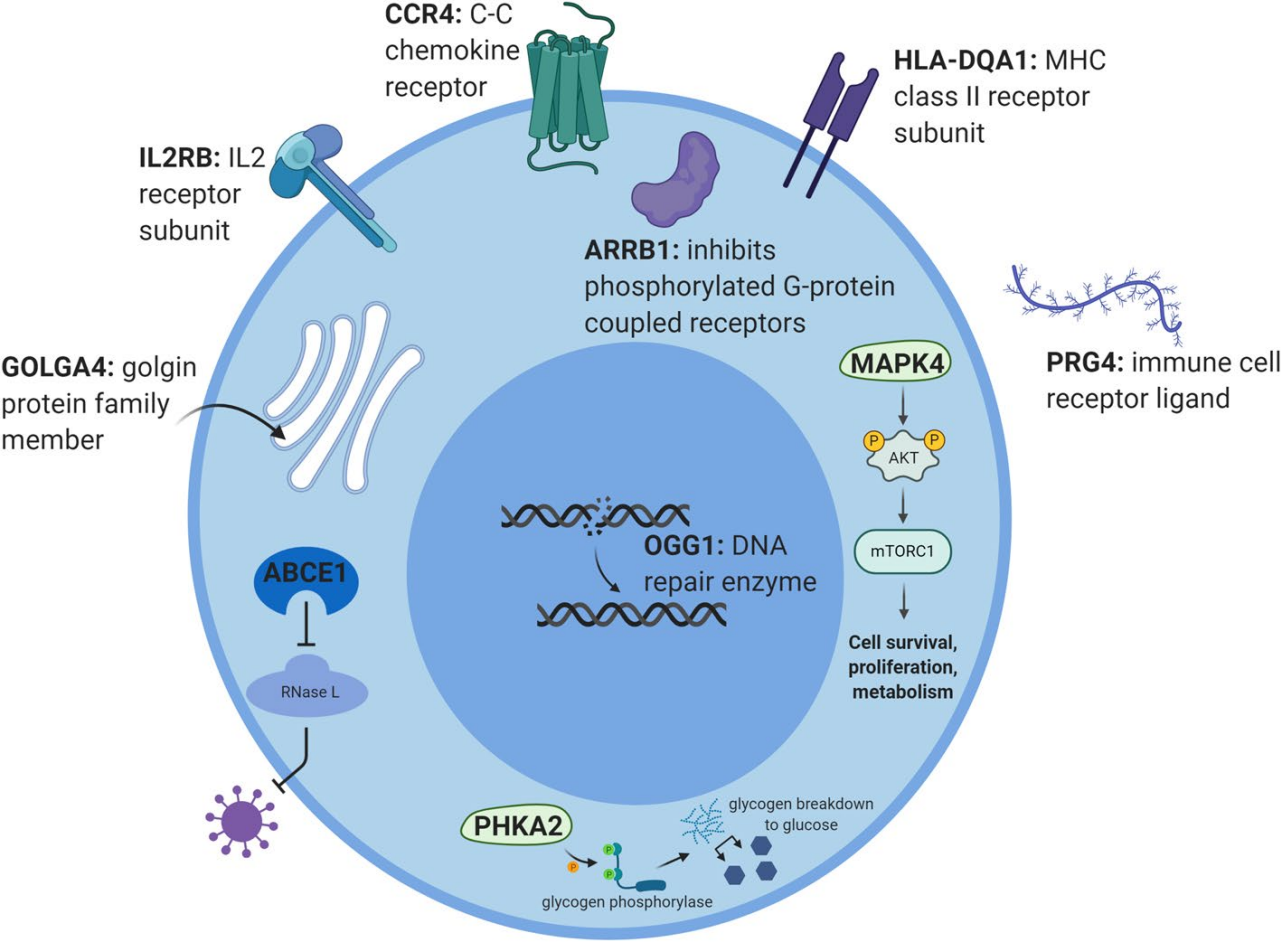
Visualization of the functions and locations of ten proteins in a hypothetical immune cell setting. All ten mRNA transcripts were downregulated in PBMCs of ME/CFS patients compared to healthy controls. Created with BioRender.com.

To put our findings in a biological context, we interrogated the existing literature on the proteins encoded by the 23 predictor genes produced by the REFS algorithm. These results were obtained in PBMCs, therefore we focused on ten proteins, MAPK4, ARRB1, GOLGA4, ABCE1, PHKA2, IL2RB, CCR4, HLA-DQA1, PRG4 and OGG1, that acted in immune pathways. The mRNA transcripts encoding these proteins were all downregulated in ME/CFS patients compared to healthy controls (Fig. 1a). Protein functions are visualized in Fig. 4 and briefly described below.

#### Intracellular signaling

In response to extracellular stimuli binding to immune cell receptors, mitogen-activated protein kinases (MAPKs) transduce intracellular signals through protein phosphorylation cascades to mediate the cellular response. MAPK4 (also known as ERK4) directly phosphorylates AKT, subsequently activating the mTOR signaling pathway and ultimately regulating cell survival, proliferation and metabolism. Decreased MAPK4 activity is associated with decreased AKT activity and proliferation^31^. Another intracellular mediator of the immune response is ARRB1 ($\beta$-arrestin 1, also known as arrestin 2). ARRB1 is found in high levels in PBLs where it inhibits G-protein coupled receptors that are phosphorylated by $\beta$-adrenergic receptor kinase. GOLGA4 (golgin A4) is a member of the golgin protein family. Upon macrophage LPS activation, formation of GOLGA4-demarked carriers is upregulated, which was found to increase TNF trafficking and subsequent secretion. Depletion of GOLGA4 consequently impairs TNF secretion^32^. ATP-binding cassette sub-family E member 1 (ABCE1), a cell membrane transporter, inhibits RNase L activity. RNase L is important in immune defense, as it degrades all RNA within the cell upon viral infection and releases antiviral IFN$\gamma$^33^. ABCE1 was downregulated in PBLs of ME/CFS patients, which correlated with upregulated RNase L^34^. Finally, PHKA2 is the hepatic isoform of the alpha subunit of phosphorylase b kinase. This enzyme phosphorylates glycogen phosphorylase b, converting it to the active glycogen phosphorylase a, which then breaks down stored glycogen to glucose. This glucose is converted through glycolysis to ATP, to meet metabolic demands. PHKA2 is thus important in providing energy to cells by maintaining glucose levels. Mutations in *PHKA2* gene cause glycogen storage disease type IXa, characterized by hypoglycemia, hepatomegaly, elevated liver enzymes, growth retardation and motor delay, hypercholesterolemia and hypertriglyceridemia. Some patients report fatigue and muscle weakness^35^.

#### Immune cell receptors and ligands

IL2RB is the $\beta$-subunit of the IL2 receptor, a receptor for IL2 that is involved in the differentiation of regulatory-, effector-, memory-, type 1 and 2 helper T cells. IL2RB-deficient patients show dysregulated IL2 and IL15 signaling, enhanced natural killer cell levels, and subsequent immunodeficiency and impaired antiviral immunity^36^. A SNP in the 3-prime untranslated region of the gene was associated with ME/CFS, interfering with miRNA binding which could reduce IL2 receptor function^37^. CCR4 (CC chemokine type 4 receptor) is a G protein-coupled receptor expressed on regulatory and type 2 helper T cells that binds CCL17 and CCL22. These chemokines cause chemotaxis of the cell, traffic leukocytes and are involved in development, homeostasis and function of the immune system^38^. Blockage of CCR4 by mogamulizumab induced fatigue in human subjects^39^. HLA-DQA1 is the $\alpha$-chain of the MHC class II receptor expressed on antigen-presenting cells such as macrophages, B lymphocytes and dendritic cells. The MHC class II receptor presents peptides to T cell receptors to activate the immune system upon viral infection. Proteoglycan 4 (PRG4) is an immune cell ligand. It is a lubricant, reducing friction between joints or boundaries, and altered expression is implicated in rheumatoid arthritis. In addition, PRG4 was found to ‘coat’ neutrophils and macrophages, perhaps by binding TLR2, -4, -5 or CD44, thereby preventing activation by low levels of pro-inflammatory cytokines. Upon inflammation, when an immune response is required, PRG4 was shed from the immune cells to allow activation^40^.

#### DNA damage repair

DNA repair enzyme 8-oxoguanine DNA glycosylase (OGG1) specifically excises the most pre-mutagenic oxidative base lesion 8-oxoguanine. Such DNA damage caused by exposure to reactive oxygen species (ROS) leads to mutagenesis or cell death. OGG1 activity prevents mutations and Alzheimer’s disease patients with an OGG1 polymorphism show increased DNA damage^41^.

## Discussion

ME/CFS is a chronic disorder characterized by persistent, disabling fatigue for which no diagnostic or prognostic test nor complete treatment is available. Several studies have sought to define biomarkers for ME/CFS by performing differential mRNA expression or DNA methylation analysis. However, as Byrnes et al.^15^ pointed out, these results were study-dependent and no definitive biomarkers were found. We used a state-of-the-art machine learning technique to distinguish ME/CFS patients from healthy controls across different platforms, several cohorts and on different levels of gene expression regulation. To our knowledge, this was the first time such a technique was used in mRNA expression data and validated in DNA methylation data.

In this study, we implemented the REFS algorithm on public mRNA expression data and found 23 genes whose changes in expression levels were able to distinguish ME/CFS patients from healthy controls. The 23 predictor genes differentiated between cases and controls with 91.57% global accuracy and returned a ROC AUC of 0.92. In addition, 48 CpG methylation sites associated with these genes were predictive of ME/CFS in four merged DNA methylation studies. Moreover, all 23 candidate genes were downregulated in ME/CFS patients while DNA methylation of almost all 48 CpG sites was enhanced. This inverse correlation between mRNA expression and DNA methylation, across different samples and studies, legitimizes the results of our study. As previously demonstrated^25^, REFS identifies a more accurate, robust gene signature than previous methods. Comparing the gene signature returned by three different methods, based on the same data, REFS outperformed both IWGCNA and univariate analysis in separating ME/CFS patients and healthy controls with a ROC AUC of 0.92. The AUC of the gene signature applied to a different platform was 0.95, and the AUC even reached 0.97 when plotting the sensitivity and specificity of the 48 predictor CpG sites.

To show the relevance of the returned predictor genes, we investigated the biological functions of ten encoded proteins active in immune pathways. This decision was based on the mRNA expression being measured in PBMCs and the literature pointing towards an important role for the immune system in ME/CFS. Sotzny et al.^42^ reviewed autoimmunity in ME/CFS, concluding that immunologic and metabolic alterations were often reported. The authors stress the potential importance of autoantibodies in the disorder and the proposed role of preceding infections. Downregulation of ABCE1, one of the encoded proteins identified in our study, concurs with the presence of previous viral infections, as the protein inhibits RNase L’s viral RNA degrading activity. Similarly, ARRB1 protein was decreased after Epstein-Barr virus-infection in mice^43^. Its downregulation in our study concurs with this finding. Recently, Mandarano et al.^44^ described evidence of immune involvement in their study of 53 ME/CFS patients. They specifically focused on T cells, showing that CD8+ T cells derived from patients had lower mitochondrial membrane potential, which points towards T cell exhaustion. PHKA2 is necessary for the first step in breaking down glycogen to glucose. Its downregulation could contribute to impaired glycolysis in immune cells. In ME/CFS, CD4+ and CD8+ T cells had impaired resting glycolysis, and plasma glucose was reduced. CD8+ T cells showed an impaired metabolic response to activation^44^. Another study found that glycogen metabolism regulates the immune functions of dendritic cells^45^. Inhibiting glycogen phosphorylase impaired their ability to produce inflammatory cytokines and stimulate T cells. These findings combined suggest that reduced PHKA2 in ME/CFS might inhibit glycogen phosphorylase activation and thus dendritic cell functioning.

Several (subunits of) immune cell receptors were also part of our gene signature and downregulated in ME/CFS. IL2RB, CCR4 and HLA-DQA1 are vital elements in proper immune response, dysregulation, whether up or down, could be evidence of a disturbed immune system or be the cause of it. The same holds true for decreased MAPK4 expression, an ubiquitous transducer of intracellular signals in response to immune cell receptor binding. Further downstream, GOLGA4 was upregulated in response to macrophage LPS activation to increase TNF secretion. TNF is the main pro-inflammatory cytokine secreted by inflammatory macrophages, and its release is important for enhancing the activation and recruitment of T cells, ensuring robust innate and adaptive immune responses. In our study however, GOLGA4 was downregulated in PBMCs of ME/CFS patients, potentially causing impaired TNF secretion and subsequently an impaired immune response to inflammation. Furthermore, decreased expression of PRG4 leads to reduced anti-inflammatory action of this protein. By binding immune cells receptors, PRG4 prevents activation by low levels of circulating pro-inflammatory cytokines. PRG4 could thus be important in low-grade inflammation causing ME/CFS^46^.

Finally, evidence has emerged that oxidative stress levels are raised in ME/CFS, for example in response to exercise, perhaps causing some of the symptoms seen in ME/CFS^47,48^. DNA damage caused by exposure to ROS leads to mutagenesis or cell death, OGG1 specifically repairs this DNA damage. OGG1 depletion in human monocyte-derived dendritic cells inhibited enhanced cell surface molecule-expression and secretion of pro-inflammatory cytokines upon exposure to 8-oxoguanine base lesions. This suggests that OGG1 is important for dendritic cell activation in response to ROS^49^. Concurrently, 8-oxoguanine base lesions did not cause acute or systemic inflammation in Ogg1-deficient mice^50^. As we found that OGG1 is downregulated in ME/CFS patients, while oxidative stress levels are increased, DNA damage might be increased, which in turn causes the release of danger associated molecular patterns (DAMPs) and activates the innate immune system. We can conclude, from the various roles of these ten genes, that their downregulation may not only contribute to immune activation, but also towards a general dysregulation of the immune response. Whether all genes are causative of the ME/CFS phenotype, or some are mere consequences of immune mayhem in ME/CFS patients remains to be investigated.

Our results return a promising gene signature for ME/CFS that needs to be validated in a well characterized clinical cohort to study its use as a diagnostic tool. In this cohort, the number of cases and controls should be balanced, as the current study suffers from cases outnumbering controls in both the mRNA expression and DNA methylation datasets. We compensated for the imbalance with stratified folds in the cross-validation. Finally, the investigation of the predictor genes has thus far been limited to a literature review. In vitro experiments with PBMCs should provide additional information regarding gene function.

To conclude, we found a mRNA expression signature of 23 genes for ME/CFS capable of separating cases and controls. These candidate genes could potentially be used as biomarkers for diagnostic purposes. In addition, ten of these genes could be interpreted in the context of a derailed immune system in ME/CFS. Those genes could be investigated further for target finding and development of future treatments for ME/CFS.

## Methods

### mRNA expression data

The mRNA expression data used in this study was retrieved as provided by Presson et al.^12^, which was made available by the CDC for the 2006 Critical Assessment of Microarray Data Analysis (CAMDA) conference (https://horvath.genetics.ucla.edu/html/CoexpressionNetwork/CFS/). We refer to this dataset as the CAMDA dataset. The CAMDA- and subsequent datasets were chosen because they are the only large, publicly available studies performed on PBMCs. PBMCs are thought to be involved in ME/CFS pathophysiology^42,44^. Included in the CAMDA dataset are mRNA expression levels in PBMCs and fatigue severity status from 118 participants. ME/CFS was determined based on the Fukuda case definition criteria^1^, and fatigue severity was estimated based on clustered scores from the SF-36 fatigue score, Multidimensional Fatigue Inventory, and Symptom Inventory Case Definition Score^12^. mRNA expression was measured on the MWG Biotech microarray platform, containing probes for approximately 20,000 transcripts.

To analyze the data, we first re-annotated the probes with HGNC gene symbols using DAVID^51^, yielding 15,419 gene-associated probes. The data was then normalized using the *Standard* scaler from sci-kit learning toolbox^27^. Samples with low fatigue severity were encoded as 0 (controls) and samples with moderate or high fatigue severity were encoded as 1 (cases), based on the clustered scores described by Presson et al.^12^. Altogether, 93 cases and 25 controls were included for REFS. A 10-fold cross-validation with stratified folds was performed to accommodate the unbalanced classes.

As a test dataset for the algorithm, mRNA expression levels in PBMCs from post-infectious, male ME/CFS patients ($n=8$) and healthy male controls ($n=7$) were included as provided by Gow et al.^13^ in the Gene Expression Omnibus (GEO)^52^ GSE14577. ME/CFS was determined with the Fukuda case definition criteria^1^ and mRNA expression was measured with Affymetrix Human Genome U133A Array. To analyze the GSE14577 data, the *series matrix* file was employed and scaled using the *Standard* scaler. Given the low number of samples, a 5-fold cross-validation was performed only for the analysis. A summary of the datasets is available in Table 2.

### DNA methylation data

DNA methylation data was retrieved from GEO (Table 2). The DNA methylation datasets pertained methylation profiles in PBMCs of ME/CFS patients and healthy controls measured with the Illumina HumanMethylation Infinium 450k BeadChip array (450k) and its successor, the Illumina HumanMethylation Infinium EPIC BeadChip array (EPIC). The 450k and the EPIC measured the methylation status of 485,577 and 865,859 CpGs, respectively, with approximately 90% of the 450k probes being present on the EPIC^53^. In all four studies, ME/CFS was determined with the Fukuda and Canadian case definitions^1,54^. From the GEO repositories, *series matrix* files containing the normalized percentage methylation per CpG were obtained. The data was pre-processed by scaling the percentage methylation per sample using the *Standard* scaler, after which the individual datasets were merged. Subsequently, the data was split for 10-fold cross-validation with stratified folds. In total, the methylation data included 149 samples (99 cases and 50 controls).

**Table 2:** Characteristics of the mRNA expression and DNA methylation datasets.

GEO accession number/name	Reference	Samples (n)	Measuring platform	Probes (n)	Type
CAMDA	Presson et al.^12^	118	MWG Biotech microarray	15,419	mRNA expression
GSE14577	Gow et al.^13^	15	Affymetrix Human Genome U133A Array	22,284	mRNA expression
GSE59489	de Vega et al.^21^	24	450k (GPL13534)	485,577	DNA methylation
GSE93266	de Vega et al.^22^	75	450k (GPL13534)	485,512	DNA methylation
GSE102504	de Vega et al.^23^	25	450k (GPL13534)	467,971	DNA methylation
GSE111183	Trivedi et al.^24^	25	EPIC (GPL21145)	866,895	DNA methylation

### REFS on mRNA expression data

**Figure 5:**
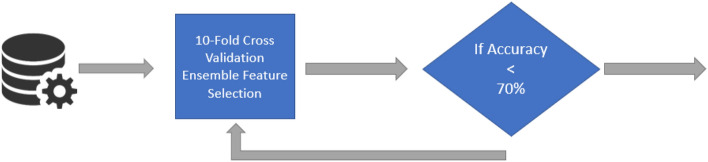
Overview of the REFS algorithm using the 10-fold global accuracy > 70% as a stop parameter.

Several studies have employed univariate feature selection, with statistic metrics such as $\chi ^2$ or $F-value$, that reduce the features using the highest scores, yielding study-specific results. To overcome this issue, REFS was used to identify a mRNA expression signature that was able to classify disease status in a PMBC study of ME/CFS patients and healthy controls. The utility and accuracy of REFS compared to other methods was previously demonstrated in miRNA and mRNA datasets^25,55^. In short, the REFS algorithm reduces the number of features to the most significant ones by combining the results of separate classifiers of distinct topologies. The algorithm gives each feature a score, based on how each different algorithm used it. In case of a tree-based algorithm, it depends on how many times the feature appeared in the tree. If it is a coefficient based algorithm, it depends on the value of the coefficient, the highest being the most important. This scoring is consistent with the Borda method^56^, the difference being that 10-fold cross validation is included for scoring. After each iteration, the most important features are selected (Fig. 5).

The REFS algorithm (Algorithm 1) was implemented on the 15,419 gene-associated probes through 10-fold cross-validation with the following classifiers: Bagging, Gradient Boosting, Random Forest, Logistic Regression, Passive Aggressive, SGD, SVC (linear), and Ridge. During the first iteration, the algorithm reduced the total features to the most significant ones, after which it reduced the number of variables by 20% per step with a stop parameter of 70% accuracy as indicated in Fig. 5. 
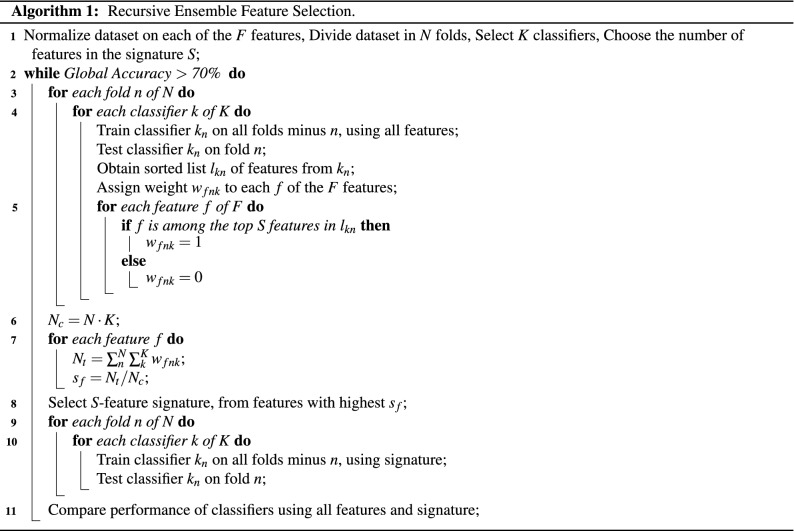


### REFS on DNA methylation data of candidate genes

**Figure 6:**
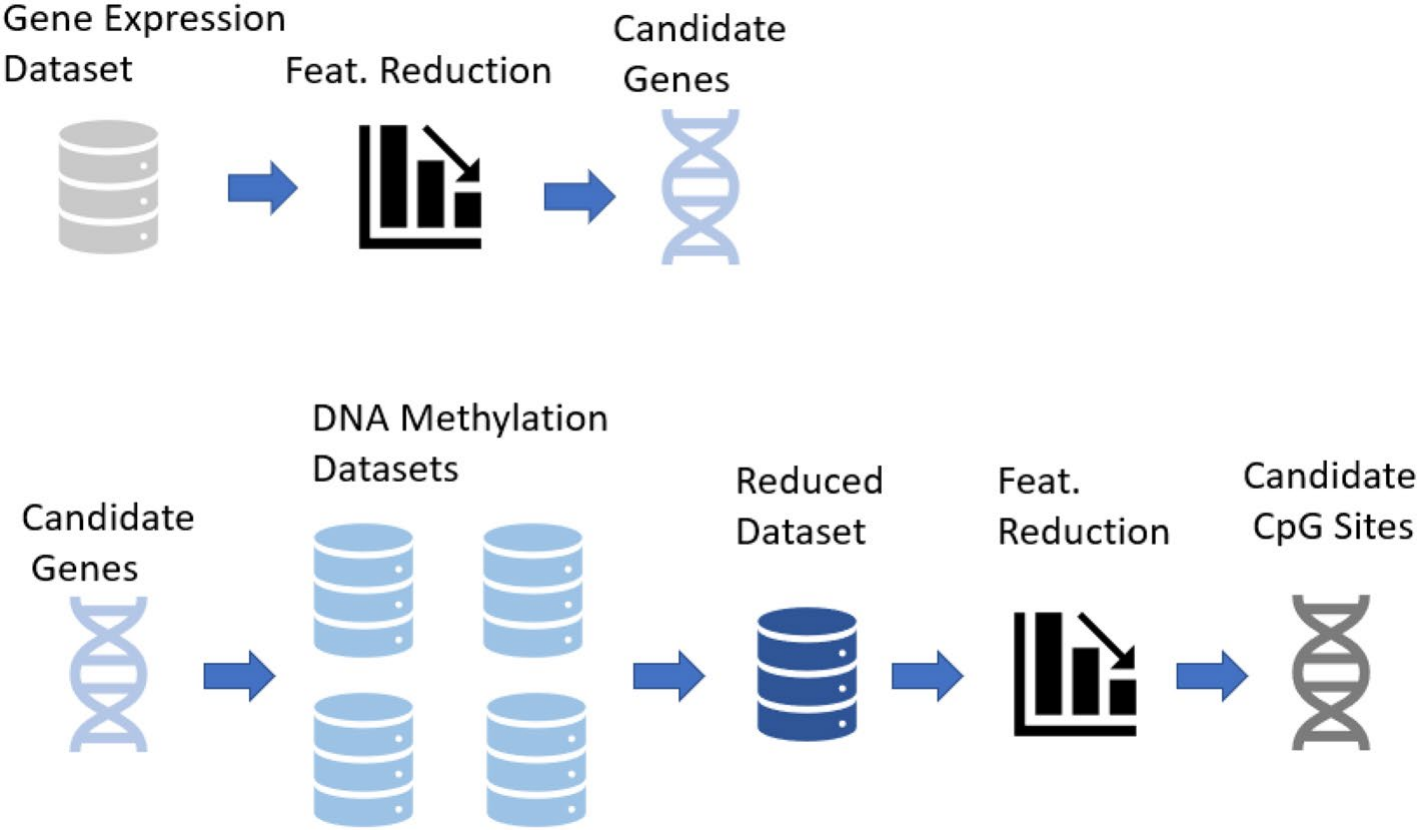
Overview of the pipeline to reduce the number of methylation data probes for REFS by selecting CpGs associated with the candidate genes.

The DNA methylation platforms were represented by a large amount of features, 485,577 and 865,859 features by the 450k and EPIC, respectively. Therefore, the search space was reduced by adopting a candidate gene approach, in which CpGs were only selected if they associated with the genes found using REFS on the CAMDA dataset. Practically, CpG probes were selected from the datasheet of the platform GPL13534 (https://www.ncbi.nlm.nih.gov/geo/query/acc.cgi?acc/=GPL13534) when the *UCSC_RefGene_Name* field matched any of the genes previously selected by the ensemble feature selection approach. This process is explained in Fig. 6.

### Biological interpretation of the mRNA expression signature

To put the genes identified with the REFS algorithm in the mRNA expression dataset in a biological perspective, the protein encoded by each gene was investigated in the existing literature available on Pubmed and Google Scholar (March 2020). Because the analyses were performed with genetic material derived from PBMCs, protein function was investigated in the context of the immune system. After initial analysis, ten proteins that functioned in immune pathways were selected for further literature review.

## Supplementary information

Supplementary information.
