# Supplementary material for: Recursive ensemble feature selection provides a robust mRNA expression signature for myalgic encephalomyelitis/chronic fatigue syndrome
Source: Sci Rep. 2021 Feb 25;11:4541. doi: 10.1038/s41598-021-83660-9 (PMC7907358; doi:10.1038/s41598-021-83660-9)
Supplement: Supplementary file 1 — Supplementary information. [file 41598_2021_83660_MOESM1_ESM.docx]

**Supplementary data**

**Supplementary Table S1.** List of 23 genes capable of distinguishing ME/CFS patients from healthy controls, found with REFS on the CAMDA dataset containing 118 samples. The protein function of ten immune-related genes was investigated in this article.

| **Gene symbol** |
| --- |
| *ABCE1* |
| *ADAM22* |
| *ARRB1* |
| *BRAT1* |
| *CCR4* |
| *COL3A1* |
| *CORO6* |
| *DENND5A* |
| *ECT2* |
| *GOLGA4* |
| *HLA-DQA1* |
| *IL2RB* |
| *KCNA2* |
| *MAPK4* |
| *NCOA6* |
| *NMNAT1* |
| *OGG1* |
| *PHKA2* |
| *PRG4* |
| *PTPRM* |
| *SPAST* |
| *STRBP* |
| *UTP4* |
